# Evaluation of miRNA-196a2 and apoptosis-related target genes: *ANXA1*, *DFFA* and *PDCD4* expression in gastrointestinal cancer patients: A pilot study

**DOI:** 10.1371/journal.pone.0187310

**Published:** 2017-11-01

**Authors:** Manal S. Fawzy, Eman A. Toraih, Afaf Ibrahiem, Hala Abdeldayem, Amany O. Mohamed, Mohamed M. Abdel-Daim

**Affiliations:** 1 Department of Medical Biochemistry, Faculty of Medicine, Suez Canal University, Ismailia, Egypt; 2 Department of Biochemistry, Faculty of Medicine, Northern Border University, Arar, Saudi Arabia; 3 Genetics Unit, Department of Histology and Cell Biology, Faculty of Medicine, Suez Canal University, Ismailia, Egypt; 4 Department of Pathology, Faculty of Medicine, Mansoura University, Mansours, Egypt; 5 Department of Pathology, Faculty of Medicine, Suez Canal University, Ismailia, Egypt; 6 Department of Medical Biochemistry, Faculty of Medicine, Assiut University, Assiut, Egypt; 7 Department of Pharmacology, Faculty of Veterinary Medicine, Suez Canal University, Ismailia, Egypt; 8 Department of Ophthalmology and Micro-Technology, Yokohama City University, Yokohama, Japan; University of South Alabama Mitchell Cancer Institute, UNITED STATES

## Abstract

Previous reports have suggested the significant association of miRNAs aberrant expression with tumor initiation, progression and metastasis in cancer, including gastrointestinal (GI) cancers. The current preliminary study aimed to evaluate the relative expression levels of miR-196a2 and three of its selected apoptosis-related targets; *ANXA1*, *DFFA* and *PDCD4* in a sample of GI cancer patients. Quantitative real-time PCR for miR-196a2 and its selected mRNA targets, as well as immunohistochemical assay for annexin A1 protein expression were detected in 58 tissues with different GI cancer samples. In addition, correlation with the clinicopathological features and *in silico* network analysis of the selected molecular markers were analyzed. Stratified analyses by cancer site revealed elevated levels of miR-196a2 and low expression of the selected target genes. Annexin protein expression was positively correlated with its gene expression profile. In colorectal cancer, miR-196a over-expression was negatively correlated with annexin A1 protein expression (r = -0.738, *p* < 0.001), and both were indicators of unfavorable prognosis in terms of poor differentiation, larger tumor size, and advanced clinical stage. Taken together, aberrant expression of miR-196a2 and the selected apoptosis-related biomarkers might be involved in GI cancer development and progression and could have potential diagnostic and prognostic roles in these types of cancer; particularly colorectal cancer, provided the results experimentally validated and confirmed in larger multi-center studies.

## Introduction

Despite an increasing number of studies unraveled the molecular mechanisms of digestive tract tumors, the clinical outcome of cancer patients is still poor with low survival rates [1]. Discovery of new biomarkers for early detection and outcome prediction of cancer is mandatory. MicroRNAs have been proposed to be novel biomarkers for human cancer [2]. These small non-coding RNAs are involved in every cellular biological process. They silence hundreds of target genes via translational repression or mRNAs degradation [3]. Several reports have suggested the significant association of miRNAs aberrant expression with tumor initiation, progression and metastasis in cancer [4], including gastrointestinal (GI) cancers [5–8].

**Fig 1:**
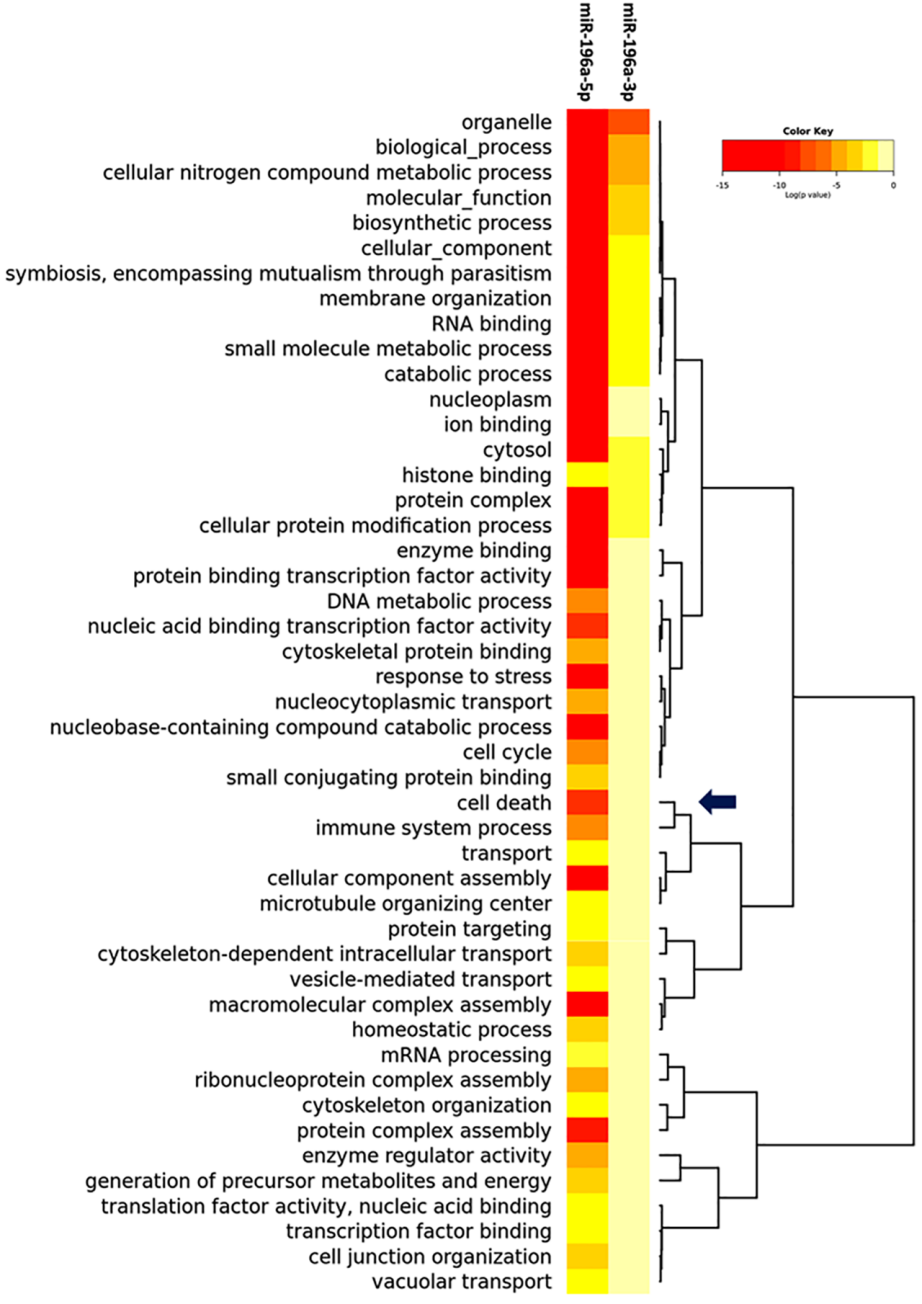
Enrichment functional analysis of hsa-miR-196a2 gene targets. Fisher's Exact test (hypergeometric distribution) was applied as an enrichment analysis method (p = 5.868485e-07). Black arrow highlighted one of the gene ontology (Cell Death) of the gene set [Data source: mirPath v.3].

Recently microRNA-196a2 (miR-196a2) gained a lot of attention [9]. It has been reported to be deregulated in various cancer types [10–12] and consequently, this up- or down-regulation may impact tumor malignancy or drug resistance according to the downstream target genes it affects. Bioinformatics analysis in our previous publication [13], has shown that miR-196a2 could target many genes enriched in cell cycle regulation, survival and apoptosis that could be involved in GI cancers. Our gene ontology (GO) analysis (Fig 1) illustrated enrollment of hsa-miR-196a2 in cell death (GO: 0008219) through targeting 81 genes including the current studied ones. In addition, it is involved in the cellular component disassembly involved in execution phase of apoptosis (GO:0006921) via targeting 11 genes including DNA fragmentation factor alpha polypeptide (*DFFA*) gene (p = 0.003670649).

One of miR-196a2-targetd genes is *Annexin A1* (*ANXA1*) which has been reported to have a vital role in cell proliferation, apoptosis, migration, and carcinogenesis [14]. Despite previous studies in the literature have evaluated its expression pattern in some GI cancers, the findings were often different. For example; it showed a higher expression level in diffuse-type gastric cancer in comparison to the intestinal type [15], high expression in esophageal adenocarcinomas correlating with advanced stage and the presence of distant metastasis [16], loss of expression in advanced gastric cancers with metastasis [17], decreased expression in gastric adenocarcinoma, but showed positive staining in advanced stage and peritoneal dissemination [18], and over-expression in both gastritis and gastric cancer [19].

Other apoptosis-related explored targets of miR-196a2 which have been detected by several bioinformatic tools which have been named in the methodology section of the current study; included *DFFA* and Programmed cell death 4 (*PDCD4*). During apoptosis, DFFA (i.e. the 45-kD subunit of DFF) is cleaved by caspase-3 that induces release and activation of its partner DFF beta (DFFB); the active component of DFF that triggers both DNA fragmentation in inter-nucleosomal chromatin regions and chromatin condensation [20]. Thus, the DFFA may play a role in malignant transformation and metastasis [21] and up- or down-regulation of its expression has been correlated with a poor prognosis in patients with esophageal carcinoma [22] and a specific pattern of apoptosis in human colonic cancer cells [23].

The PDCD4 is a neoplastic transformation inhibitor that has been reported to bind to the “eukaryotic translation initiation factor 4A1” leading to translation inhibition [24]. Its loss of expression has been associated with malignant transformation in gastric cancer [25] and Helicobacter pylori induced epithelial-mesenchymal transition in gastric cancer cell lines [26]. In addition, it has been reported to be related to the tumor differentiation in digestive tract cancers [27].

The three selected targets in the current study were experimentally validated by high-throughput methods in cancer cell lines in prior studies (Table 1).

**Table 1:** Summary of previously published validation studies for the current miR-196a2 putative targets.

Target	Methods	Tissue	Cell line	Score	Validation type	Publication	PMID	Source
**ANXA1**	RA	Mammary gland	MCF7	--	Direct	Luthra et al., 2008 [28]	18663355	TarBase 6.0
	PAR-CLIP	Kidney	HEK293	0.517	Direct	Kishore et al., 2011 [29]	21572407	TarBase 7.0
	PAR-CLIP	Cervix	TZMBL	0.517	Direct	Whisnant et al., 2013 [30]	23592263	TarBase 7.0
**PDCD4**	PAR-CLIP	Kidney	HEK293	--	Unknown	Hafner et al., 2010 [31]	20371350	miRTarBase
**DFFA**	PAR-CLIP	Lymphoma	BC1	0.818	Direct	Gottwein et al., 2011 [32]	22100165	TarBase 7.0
	PAR-CLIP	Kidney	HEK293	0.818	Direct	Kishore et al., 2011 [29]	21572407	TarBase 7.0
	PAR-CLIP	Kidney	HEK293	0.818	Direct	Kishore et al., 2011 [29]	21572407	TarBase 7.0
	PAR-CLIP	Kidney	HEK293	0.818	Unknown	Hafner et al., 2010 [31]	20371350	miRTarBase

RA: Luciferase reporter gene assay; PAR-CLIP: photoactivatable ribonucleoside-enhanced Cross-linking and immunoprecipitation.

Evaluation of the tissue-specific miRNA and the related target-gene expression levels in cancer may improve our understanding of their role as diagnostic and/or prognostic biomarkers [33]. Therefore, the current study aimed to investigate the expression levels of miR-196a2, three of its putative targets; *ANXA1*, *DFFA* and *PDCD4* mRNAs and annexin A1 protein in GI cancer tissue samples compared to cancer-free tissues. The possible relationship between the aforementioned expression levels and both the clinicopathological features and the patient's prognosis, in addition, have been tested.

## Materials and methods

### Ethical approval and informed consent

The study was conducted in accordance with the guidelines in the Declaration of Helsinki and it has been approved by the Medical Research Ethics Committee of Faculty of Medicine, Suez Canal University (Approval No. 2774). All participants have provided written consent.

### Study participants and specimen collection

Fifty-eight archived formalin-fixed paraffin embedded (FFPE) specimens for cancer patients with digestive tract tumors who underwent radical biopsy have been collected from Pathology laboratory of Mansoura Oncology Center, Mansoura and Pathology laboratory of the Suez Canal University Hospital, Ismailia, Egypt, dating back for 4 years. Baseline characteristics of the study groups are illustrated in Table 2. FFPE cancer specimens included oesophageal cancer (n = 10), gastric carcinoma (n = 14), small intestine cancer (n = 7), and colorectal cancer (n = 27).

**Table 2:** Baseline characteristics of cancer patients.

	EC	GC	SIC	CRC
**Number**	10	14	7	27
**Age, y**				
Mean ± SD	50.3 ±11.5	406 ±10.5	54.8 ±11.5	52.7 ± 11.4
Range	32–70	29–59	37–67	33–72
<40y	2(20)	9(64.3)	1(14.3)	7(25.9)
≥40y	8(80)	5(35.7)	6(85.7)	20(74.1)
**Gender**				
Female	3(30)	8(57.1)	4(57.1)	7(25.9)
Male	7(70)	6(42.9)	3(42.9)	20(74.1)
**Tumor grade**				
Grade 1	4(40)	5(35.7)	5(71.4)	10(37)
Grade 2	6(60)	2(14.3)	1(14.3)	8(29.6)
Grade 3	0(0)	7(50)	1(14.3)	9(33.3)
**Tumor size**				
T1	0(0)	1(7.1)	0(0)	6(22.2)
T2	6(60)	8(57.1)	2(28.6)	5(18.5)
T3	4(40)	3(21.4)	5(71.4)	13(48.1)
T4	0(0)	2(14.3)	0(0)	3(11.1)
**LN infiltration**				
N0	9(90)	8(57.1)	3(42.9)	19(704)
N1	1(10)	4(28.6)	4(57.1)	8(29.6)
N2	0(0)	2(14.3)	0(0)	0(0)
**TNM Stage**				
Stage 1	5(50)	5(35.7)	2(28.6)	8(29.6)
Stage 2	5(50)	7(50)	3(42.9)	10(37)
Stage 3	0(0)	2(14.3)	2(28.6)	9(33.3)

Data are presented as number (percentage), mean ± standard deviation, and range (minimum-maximum). EC, esophageal cancer; GC, gastric carcinoma; SIC, small intestine cancer; CRC, colorectal cancer; TNM, Tumor size, Lymph node, and Metastasis staging system.

All patients were confirmed histologically to have primary tumors without receiving a chemotherapy or radiotherapy prior to the surgical intervention. Tumor specimens were re-cut and examined via two independent pathologists with a 100% rate of concordance. Hematoxylin and Eosin (H&E) stained slides were reviewed to confirm the diagnosis and for identification of their morphological criteria, pathological grade [34] and TNM staging (tumor size, lymph node involvement, and distant metastasis) system [35]. The medical records of the patients were reviewed to assess the clinical data; age, sex, and the staging. Sections of cancer–free tissues adjacent to the tumors were cut, examined, and collected to serve as controls during the genetic profiling.

### RNA extraction

Total RNA, including small RNAs, was isolated from FFPE tissue sections using the Qiagen miRNeasy FFPE Kit (Qiagen, 217504) following the manufacturer's instructions. NanoDrop ND-1000 spectrophotometer (NanoDrop Tech., Inc. Wilmington, DE, USA) has been used for RNA concentration and quality determinations [36].

### miRNA expression analysis

The complementary DNA (cDNA) was prepared from total RNA using TaqMan MicroRNA Reverse Transcription (RT) kit (P/N 4366596; Applied Biosystems, Foster City, CA, USA), miRNA-196a2 specific stem–loop primer (assay ID 478230_mir), and the endogenous control RNU6B primer (assay ID 001093) as previously described in details [13].

### Gene targets selection and mRNA expression profiling

Three putative apoptosis-related targets (*ANXA1*, *DFFA*, and *PDCD4*) were reported to be targeted by miR-196a2-5p using DIANA-TarBase version 7.0 (http://diana.cslab.ece.ntua.gr/tarbase/) which are manually curated and based on high throughput experiments [37]. The bioinformatic algorithms microRNA.org [38], miRTarBase [39] and miRNAmap [40] were used to demonstrate miR-196a2 binding sites in the ANXA1, DFFA, and PDCD4 mRNAs. Extracted mRNAs were converted to cDNA using “High Capacity cDNA Reverse Transcription Kit (Applied Biosystems, P/N 4368814)” and quantified via Real Time PCR technology system using the TaqMan^®^ assay (Applied Biosystems, assay ID Hs00167549_m1 for ANXA1, Hs00189336_m1 for DFFA, and Hs00377253_m1 for PDCD4), TaqMan^®^ Endogenous control assay for GAPDH (Applied Biosystems, catalogue no Hs402869), and (2×) Taqman^®^ Universal PCR master mix II, No UNG (Applied Biosystems, P/N 4440043). The PCR was performed according to the "Minimum Information for Publication of Quantitative Real-Time PCR Experiments" guidelines on StepOne™ Real-Time PCR System (Applied Biosystem) as follows: 95°C for 10 min followed by 40 cycles of 92°C for 15 seconds and 60°C for 1 min. Randomly selected study samples (10%) were retested in separate runs to evaluate the reproducibility of the results which showed very close quantitative cycle (Cq) values and low standard deviations. Gene expression was calculated using relative quantification method [41].

### Annexin A1 protein expression by immunohistochemistry

Paraffin-embedded histological sections (4 μm) were deparaffinized and rehydrated in a graded series of ethanol. Endogenous peroxidase activity was blocked with 0.3% hydrogen peroxide in methanol for 60 min. Following heat-induced epitope retrieval at 37°C for 30 min, the tissue sections were incubated with a monoclonal anti-human annexin A1antibody (1:50 Monoclonal mouse IgG1 Clone No. 686106) overnight at 4°C. After washing with phosphate-buffered saline (PBS), sections were incubated with secondary antibodies for 20–30 min at room temperature. Finally, diaminobenzidine (DAB) substrate (CELL MARQUE, cat. No. 957D-30, 1:1 DAB chromogen cat. No. 957D-31 and DAB buffer substrate cat. No. 957D-32) was used for visualization. Then, the slides were washed thoroughly in distilled water, counterstained with hematoxylin and mounted on glass slides. Negative controls were obtained by using mouse IgG instead of primary antibody. The intensity and extent of the immunostaining were scored independently by two pathologists blinded to the patients' information with the resolution of disagreements by consensus.

The expression of annexin A1 was considered positive if the nucleus and/or cytoplasm of cancerous and normal tissue indicated at least 5% dye-affinity when the total field of view was observed at x40 magnification. Positive stromal staining was not included in the evaluation. In the scoring of annexin A1 protein expression levels, the extent and intensity of positive staining were considered. The extent of positivity was scored according to the percentage of cells showing positive staining, as follows: 0, <5% positive; 1, 5–25% positive; 2, 26–50% positive; and 3, >50% positive. Staining intensity was scored as follows: “0 (−, no staining); 1 (+, weak staining); 2 (++, moderate staining); and 3 (+++, strong staining)”. The final staining scores of annexin A1 were analyzed by multiplying the extent of positivity score and the staining intensity score, yielding a total score ranging between 0 and 9. Optimal cut off values were identified as follows: a staining index score of ≥ 4 defined a tumor as positive (high expression), and a staining index score of < 4 defined a tumor as annexin A1 negative (low expression) [42].

### In silico data analysis

Gene and protein data were retrieved from the GeneCards Human Gene Database “(http://www.genecards.org/)” and the Ensembl Genome Browser “(http://www.ensembl.org/)”. The STRING database “(Search Tool for the Retrieval of Interacting Genes/Proteins)” version 10.0 was used (http://string-db.org/) to reveal known and predicted protein-protein interactions between the selected target genes with others, including direct (physical) and indirect (functional) associations [43]. STRING and DIANA-miRPath v2.0 web-servers [44] were used to analyze gene ontology terms of biological process, molecular function, cellular compartment, and KEGG enrichment pathways of miR-196a2 target genes. The selected gene targets in particular shared the same GO function regarding apoptosis.

### Statistical analysis

Data were managed using R language version 3.3.1 (corrplot, dplyr, and ggplot2 packages) and the SPSS for windows software (version 22.0). Chi-square (χ^2^), Mann-Whitney U (MW) and Kruskal-Wallis (KW) tests were used for comparison as data were not normally distributed. A two-tailed *P*-value of < 0.05 was considered statistically significant. The fold change of miRNA expression in each patient cancer tissue sample relative to the corresponding non-cancer tissue of the same sample was calculated via Livak method based on the threshold cycle (C_T_) value with the following equation: relative quantity = 2^−ΔΔ*CT*^ [41].

## Results

### miR-196a2 and the selected genes expression profile in cancer

**Fig 2:**
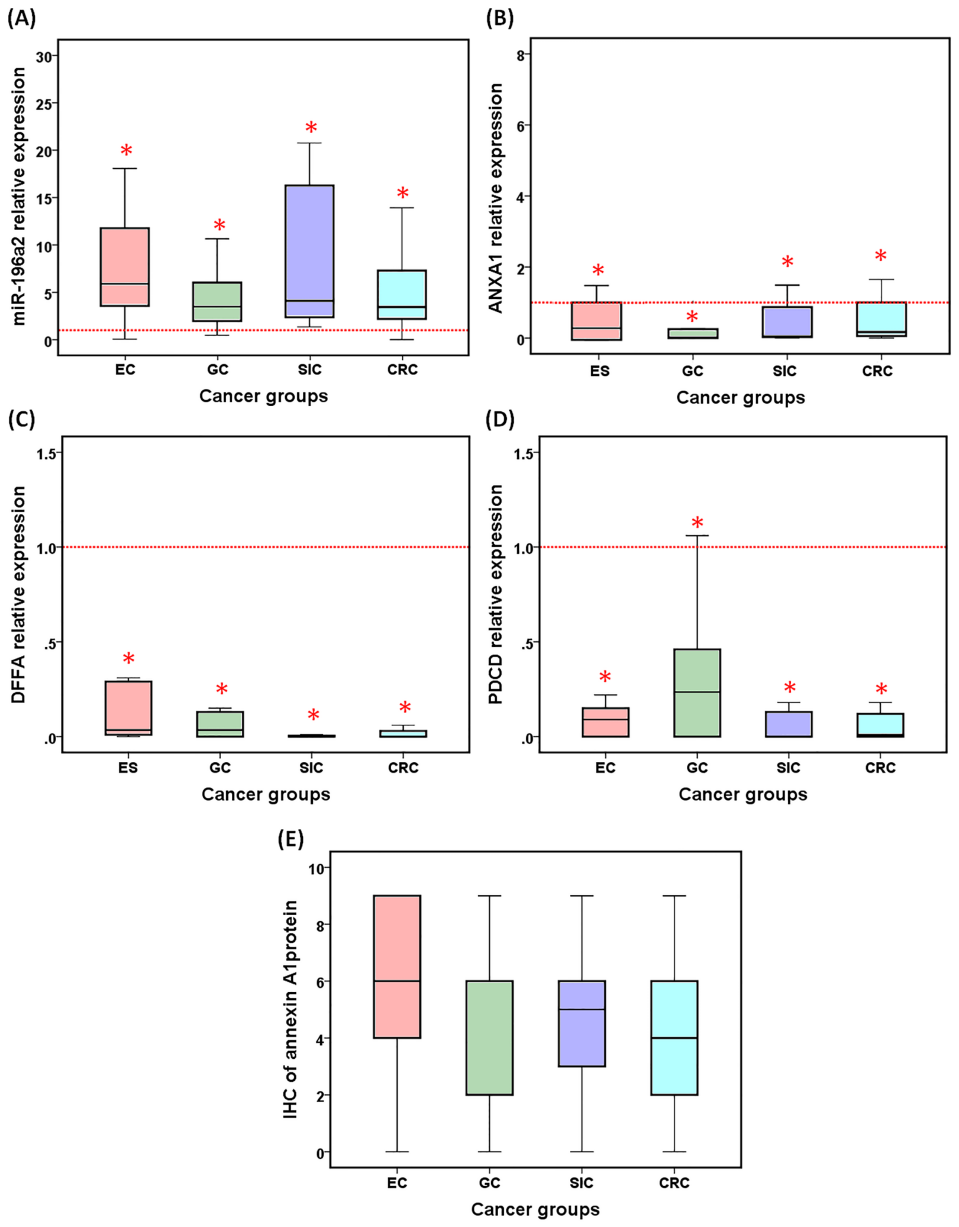
Relative expression of miR-196a2 and targets in cancer tissues compared to control. (A-D) Box plots represent median (quartiles) of gene fold change stratified by cancer type, calculated using LIVAC method. EC, esophageal cancer; GC, gastric carcinoma; SIC, small intestine cancer; CRC, colorectal carcinoma; red dotted lines are equivalent for relative expression at 1.0 of controls. Mann-Whitney U test was used. Statistical significance at *P*<0.05. (E) Annexin A1 protein expression assessed by immunohistochemistry (IHC).

Our results revealed significant elevated levels of miR-196a2 and low expression of the selected genes (*ANXA1*, *DFFA*, *PDCD4*) in all groups of GI cancer patients (Fig 2). Over-expression of miR-196a2 was observed in 80%, 86%, 100%, and 89% in EC, GC, SIC, and CRC, respectively. Similarly, down-regulation of selected genes were noticeable in a vast majority of cancer tissue specimens.

### Correlation analysis between annexin A1 protein and *ANXA1* gene expression profile

**Fig 3:**
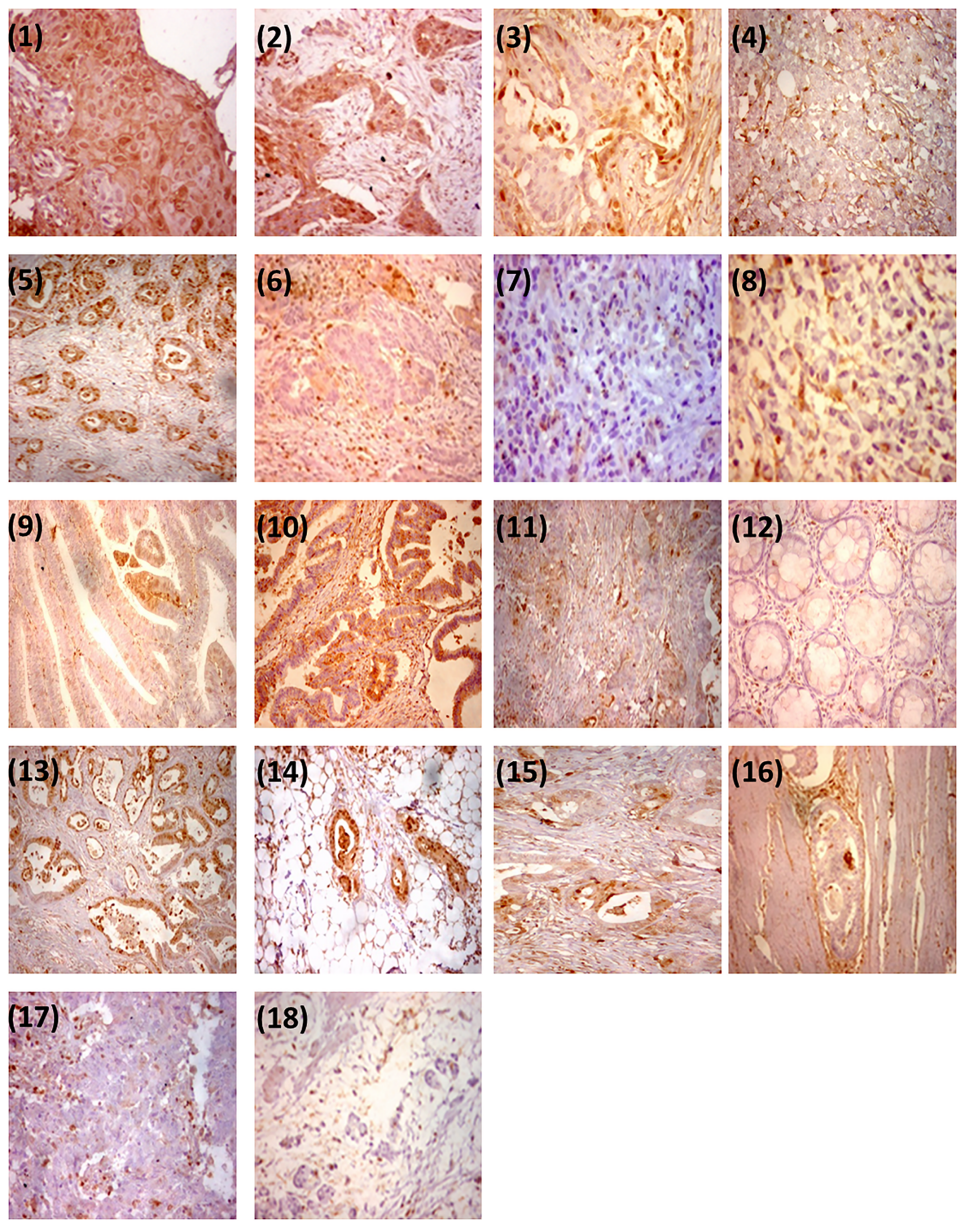
Immunohistochemical analysis of annexin A1 protein expression in GI cancer. (1) Normal esophageal mucosa normally express annexin A1 nuclear and cytoplasmic (x 200). Esophageal GII squamous expressed annexin A1 (nuclear) with score 4 (2x200); Esophageal adenocarcinoma grade II expressed annexin A1 (nuclear) with score 3 (3x200); while grade III esophageal adenocarcinoma with score 6 (4 x100). Gastric adenocarcinoma grade I expressed annexin A1 with score 9 (5x100); grade II expressed it with score 3 (6 x200); while Undifferentiated gastric carcinoma, and gastric signet ring carcinoma (8) didn’t express annexin A1 (score 0) (7 x200) (8 x200) respectively; Small intestinal villous adenoma express annexin A1 nuclear and cytoplasmic with score 3 (9 x200); Small intestinal adenocarcinoma grade I express annexin A1 cytoplasmic and nuclear with score 6 (10 X200); while lesser expression of annexin A1 score 3 was detected in small intestinal adenocarcinoma grade II (11 x200). Normal colonic mucosa didn’t express annexin A1 (score 0) (12 X100); Colonic adenocarcinoma grade I expressed annexin A1 with score 9 (13x100); Colonic adenocarcinoma grade I infiltrating subserosal fat expressed annexin A1with score 9 (14x100); Colonic adenocarcinoma grade II expressed annexin with score 4 (15x200); (16) Colonic adenocarcinoma grade II infiltrating muscle and didn’t express annexin A1 score 0 (annexin 16 x200); Colonic adenocarcinoma grade III and colonic mucinous carcinomas expressed annexin A1 in less than 5% of tumor score 0 (17 x200); (18 x100), respectively.

Immunohistochemistry staining of annexin A1 protein is shown in Fig 3. Annexin A1 protein expression was positively correlated with its gene expression profile; correlation coefficients were r = 0.786, *P* = 0.007 in esophageal cancer, r = 0.689, *P* = 0.006 in gastric carcinoma, r = 0.739, *P* = 0.058 in small intestine carcinoma, and r = 0.470, *P* = 0.013 in colorectal carcinoma.

### Correlation analysis between miR-196a2 and its selected targets

**Fig 4:**
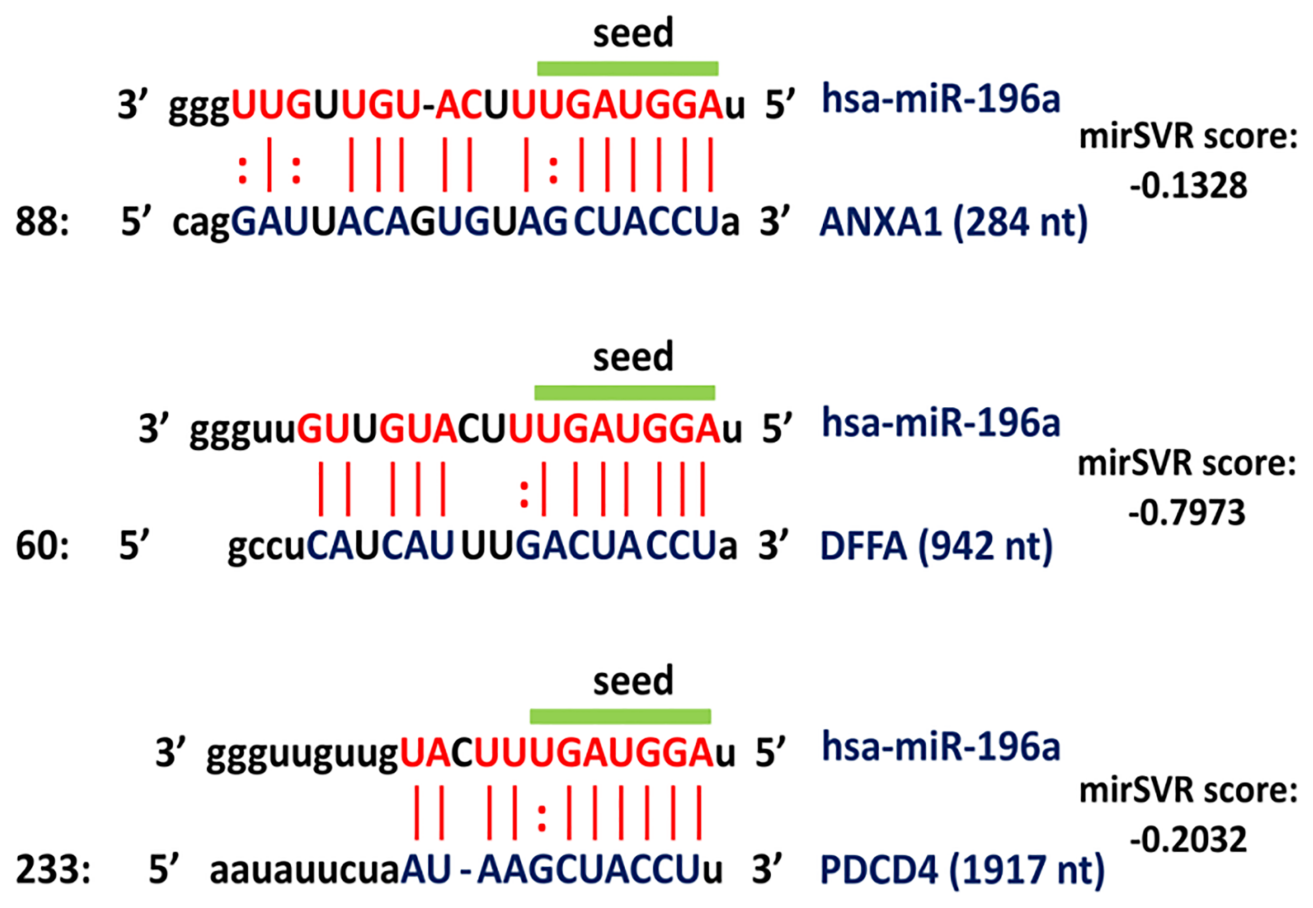
Predicted miRNA: Gene interactions. The predicted miRNA (red color)::mRNA (blue color) duplex. The putative target sites predicted by miRanda are scored for likelihood of mRNA down-regulation using mirSVR, a regression model that is trained on sequence and contextual features of the predicted miRNA::mRNA duplex. The colon (:) represents the location of a G/U wobble base pairing [Data source: microRNA.org].

**Fig 5:**
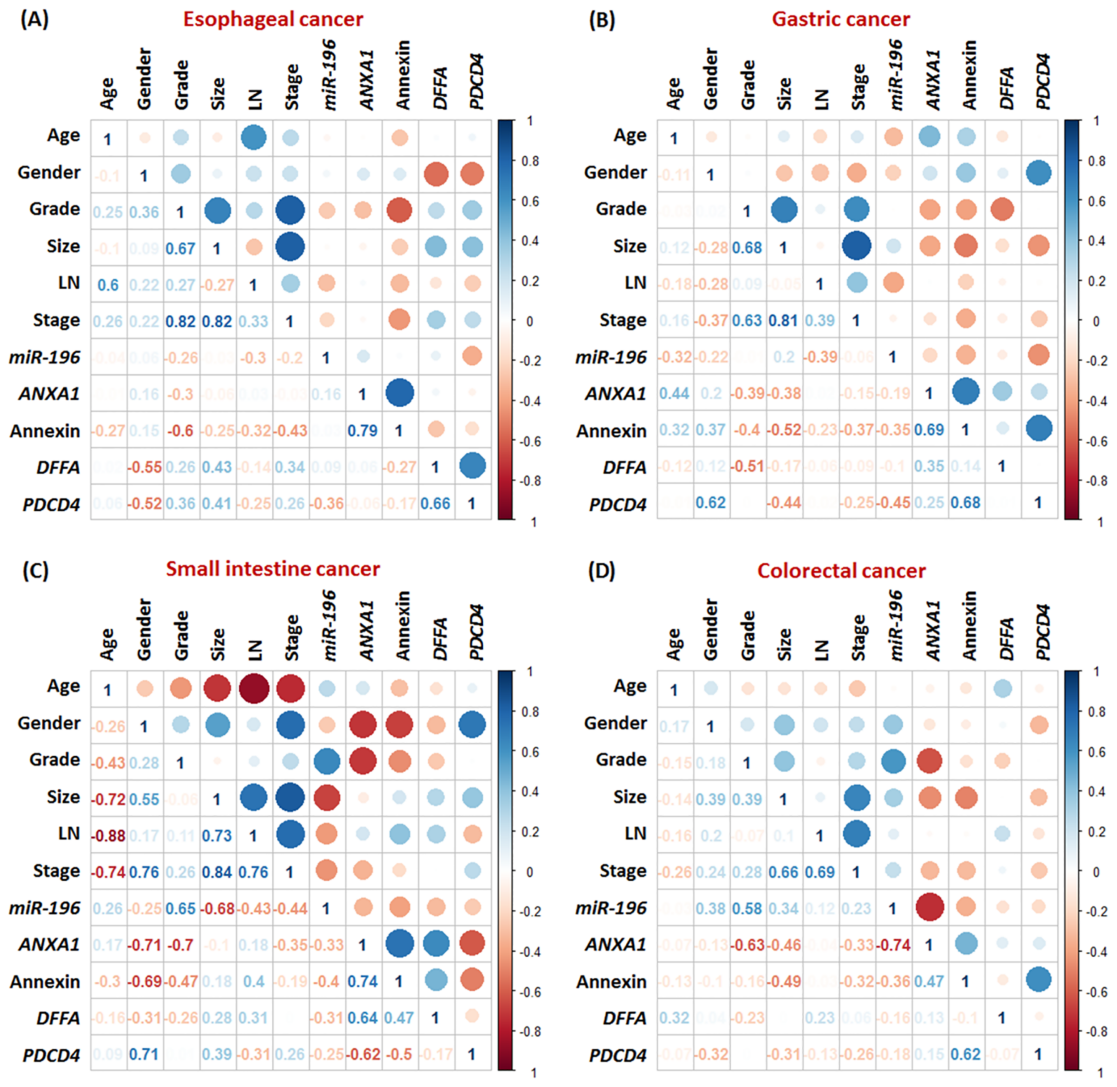
Correlation analyses of miR-196a2 and its targets with clinicopathological features. Pearson's correlation was performed by corrplot R package. The intensity of the color in the right bar indicates the degree of correlation.

The interaction between miR-196a2 with target genes showed good base pairing at the seed sequence (nucleotide 2–8), the central region and the 3' end of the miRNA::ANXA1 duplex (canonical sites), with little 3' pairing in miR-196a2::DFFA and miR-196a2::PDCD4 duplexes (seed sites) (Fig 4). In colorectal cancer, over-expression levels of miR-196a were negatively correlated with annexin A1 protein expression (r = -0.738, *P* < 0.001). However, correlation analyses revealed no significant association of miR-196a2 with any other mRNA targets in GI tumors. Correlated expression between gene targets was observed in various tumor types. Strong to moderate positive correlation was observed between *PDCD4* and *DFFA* in esophageal cancer tissues (r = 0.657, *P* = 0.039), and between *PDCD4* and *ANXA1* in gastric carcinoma (r = 0.683, *P* = 0.007) and colorectal cancer (r = 0.617, *P* = 0.001) (Fig 5).

### An association of miR-196a2 and the selected target expression profiles with the clinicopathological features

**Fig 6:**
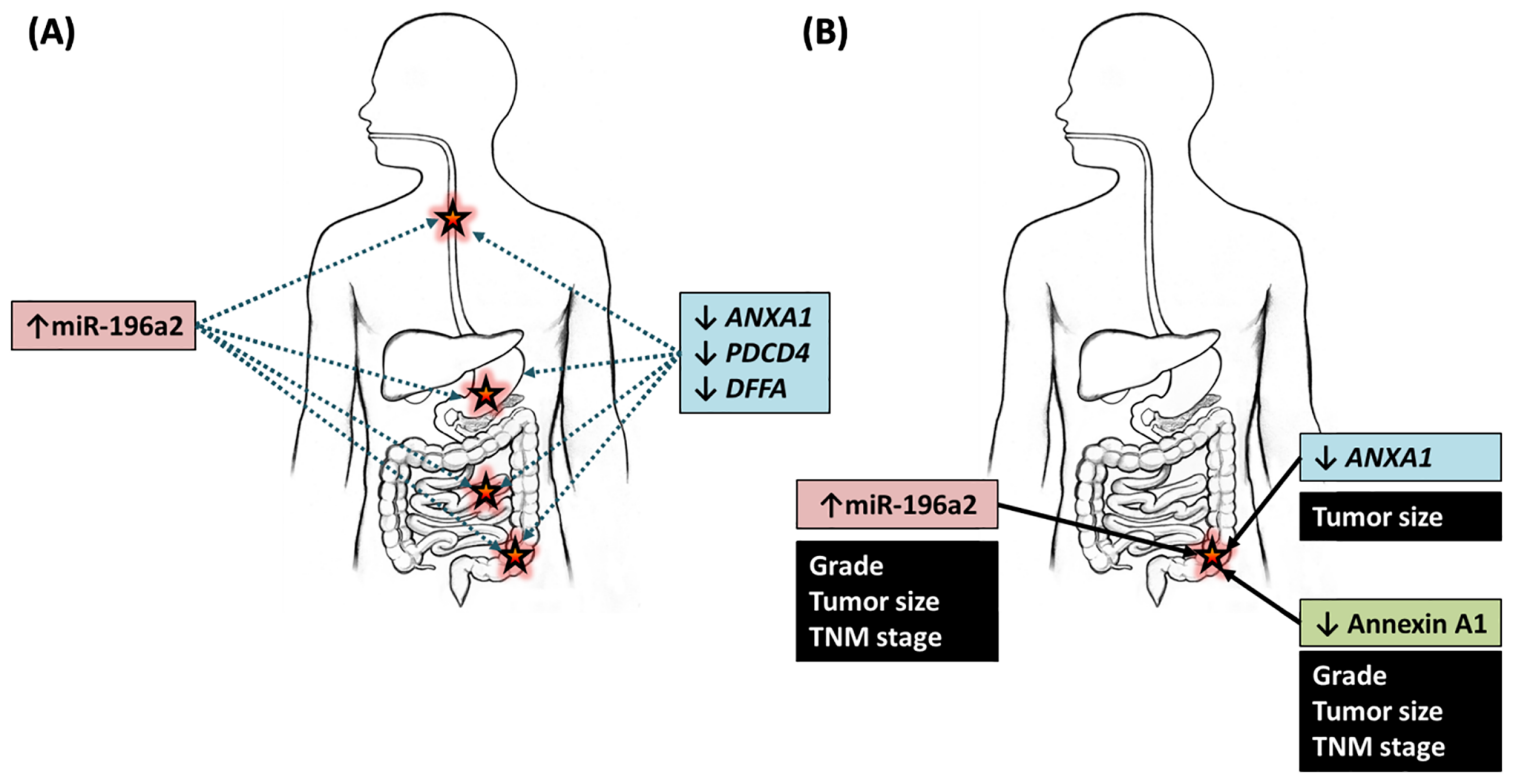
Summary of the role of miR-196a2 and the selected targets in gastrointestinal tumors of the current study. As a diagnostic (A) and prognostic (B) tumor markers in esophageal, gastric, small intestine, and colorectal cancer.

Stratified analysis by cancer site revealed that miR-196a2, *ANXA1* gene, and annexin A1 protein were indicators of poor prognosis in colorectal cancer (Fig 6). Up-regulation of miR-196a2 and down-regulation of annexin A1 were associated with poor differentiation, larger tumor size, and advanced clinical stage. In addition, lower levels of *ANXA1* showed association with larger tumors (*p* = 0.020), S1 Table.

### In silico network analysis

The major biological process, molecular function, cellular components and KEGG pathways mostly related to *ANXA1*, *DFFA*, and *PDCD4* are illustrated in S2 Table. They play key roles in vital cellular processes involved in tumorigenesis, including microRNAs in cancer [KEGG hsa05206] and apoptosis [KEGG hsa04210] pathways.

**Fig 7:**
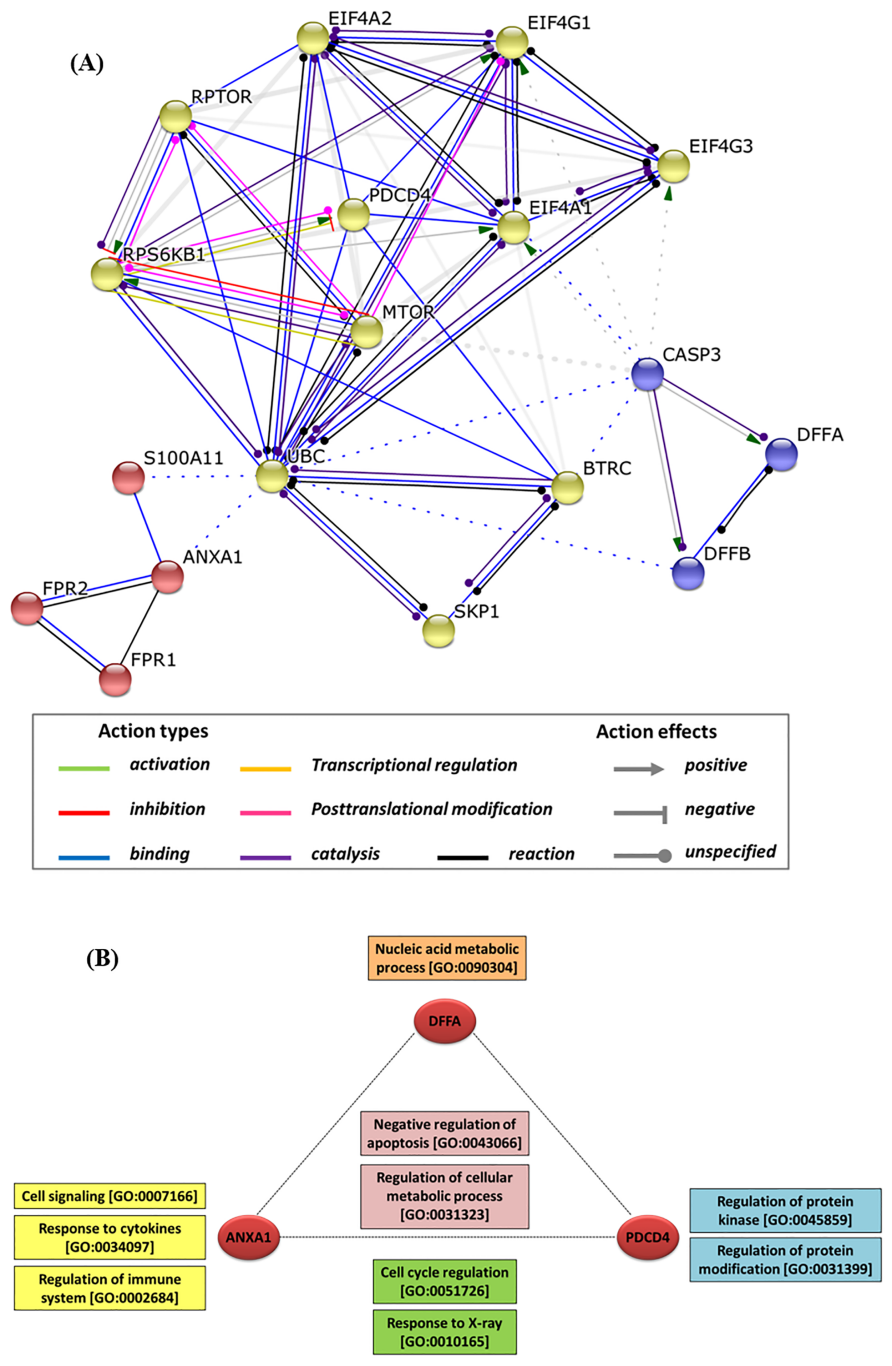
STRING analysis of miR-196a2 targets. (A) Protein-protein interaction network. Network nodes represent proteins, target genes are encircled in red, edges represent protein-protein associations, colored according to action types between proteins. The network of the three target genes (*ANXA1*, *PDCD4*, and *DFFA*) composed of 18 nodes and 57 edges with clustering coefficient of 0.803. (B) Enrichment analysis showing gene ontology of biological processes. Red nodes are the target genes, yellow box specific for *ANXA1*, blue for *PDCD4*, orange for *DFFA*, green; function shared by *ANXA1* and *PDCD4*, and pink for biological processes implicated by the three genes.

STRING analysis showing the network interaction between miR-196a2 target proteins is demonstrated in Fig 7. In the first cluster, ANXA1 protein can bind both formyl peptide receptors (FPR1 and FPR2) which are powerful neutrophils chemotactic factors and ubiquitin C (UBC) involved in various cellular activities as “protein degradation, DNA repair, cell cycle regulation, kinase modification, endocytosis, and regulation of other cell signaling pathways”. The second cluster showed that DFFA protein can form a complex with DNA fragmentation factor-beta subunit (DFFB) that induces “DNA fragmentation and chromatin condensation during apoptosis”. It can also bind to caspase 3 (CASP3), “apoptosis-related cysteine peptidase”; involved in other caspases activation cascade responsible for execution of apoptosis. Finally, in the third module, PDCD4 inhibits translation initiation by hindering the interaction between eukaryotic translation initiation factors EIF4A1 and EIF4G required for cap recognition and mRNA binding to the ribosome.

## Discussion

In our prior pilot study, we investigated circulatory and tissue miR-196a2 levels in various types of solid tumors [13]. In the current study, gastrointestinal cancer patients showed tissue miR-196a2 over-expression and down-regulation of three apoptosis-related genes *ANXA1*, *DFFA*, and *PDCD4*. These three genes were reported and validated in previous studies to be targeted by miR-196a2-5p using different computational tools. Disturbances in the apoptotic pathway promote uncontrolled cell growth, which is a key event in tumor development and metastasis [45]. Similar to the current findings, high expression levels of miR-196a2 were also reported in several human tumors [13], including esophageal carcinoma [46], gastric cancer [10], pancreatic adenocarcinoma [47], colorectal cancer [11], and hepatocellular carcinoma [12]. Several genetic and epigenetic mechanisms regulate the miRNA processing and expression. Deregulation could be caused by chromosomal abnormalities, single nucleotide polymorphisms (SNPs), epigenetic changes, and defects in miRNA biogenesis pathways [36]. MiR-196a2 resides in a particular genomic region (12q13.13) that is prone to somatic mutations and translocations, and rendered susceptible to oral cavity cancer, esophageal cancer, leukemia, medulloblastoma, and breast cancer (http://www.cancerindex.org/geneweb/clinkc12.htm). In a genome-wide association study (GWAS), Spain *et al*. [48] have found 12q13.13 genomic region to be associated with colorectal cancer. Furthermore, within MIR-196A2 gene, a genetic variant rs11614913 (C/T) was identified in the stem region of the secondary precursor that have a significant impact on miRNA expression [49–51]. Recently, in breast cancer tissues, Zhao *et al*. [52] reported higher risk of gaining somatic mutations during malignant transformation among C allele carriers of that particular SNP (rs11614913). Interestingly, the MIR-196A2 gene is located near the fragile site *FRA12A* at 12q13 [53]. Fragile sites are considered preferential sites of “sister chromatid exchange, deletion, amplification, translocation, or insertion” of tumor-associated viruses. Another putative mechanism for aberrant miRNA expression could be altered epigenetic regulation of the transcriptional regulatory sequences. Silencing of the CpG island upstream of the MIR196A2 gene by hypermethylation was associated with reduced breast cancer risk, and DNA hypomethylation of the gene was associated with over-expression in various tumor types [50, 54].

Additionally, the current results revealed that miR-196a2, *ANXA1*, and annexin A1 deregulation were associated with unfavorable prognosis in colorectal carcinoma patients. Tumor specimens with up-regulated miR-196a2 and down-regulated annexin A1 showed high pathological grade, larger tumor size, and advanced stage. In agreement with other previous studies, miR-196a2 over-expression was correlated with aggressive progression and reduced survival in colorectal patients [11] and pancreatic cancer [47]. Therefore, miR-196a2 is considered an oncogenic microRNA (oncomir) given its abilities to suppress the action of several apoptotic and tumor suppressor genes, and to promote cell proliferation, invasion and metastasis [13]. Our *in silico* analysis revealed that miR-196a2 was implicated in several cancer-related KEGG pathways. It can target transcriptional dysregulation in cancer [hsa05202], pathways in cancer [hsa05200], and cell cycle pathway[hsa04110] via silencing *CCND2* (Cyclin-D2), *CDKN1B* (cyclin-dependent kinase inhibitor 1B), *IGF1R* (insulin like growth factor 1 receptor), *SMC3* (structural maintenance of chromosomes 3), *ESPL1* (extra spindle pole bodies like 1), *ITGAV* (integrin subunit alpha V), and *BMP4* (bone morphogenetic protein 4). Similarly, miR-196a2 exerts an oncogenic effect in head and neck cancer cells by *ANXA1* suppression and radio-resistance enhancement [55], aggravates cell proliferation and metastasis in gastric cancer by targeting p27kip1 and radixin genes [56, 57], promotes colorectal cell carcinoma detachment, migration, invasion and chemo-sensitivity towards platin derivatives via regulating *HoxA7* (a subset of homeotic genes), *HoxB8*, *HoxC8* and *HoxD8* [58], enhances tumor progression in pancreatic cancer through the regulation of nuclear factor kappa-B-inhibitor alpha [59], promotes cell proliferation and invasion in non-small cell lung cancer via silencing *HOXA5* [60], and potentiate an oncogenic phenotype in cervical cancer cells by negative regulation of *netrin 4*, *FOXO1* (forkhead box o1), and *p27kip1* [61, 62].

Our data showed that *ANXA1* gene and protein were down-regulated in all GI cancers, and was associated with high pathological grade, larger tumor size, and advanced stage in colorectal carcinoma. Loss of function or expression of *ANXA1* gene has been detected in multiple human cancers; as esophageal squamous cell carcinoma [63, 64], gastric cancer [65], thyroid cancer [66], head and neck cancer [67], prostate cancer [68], endometrial carcinoma [69], and B-cell non-Hodgkin's lymphomas [70]. In line with our observation, *ANXA1* mRNA and protein expressions were markers of differentiation in squamous cell carcinoma of the cervix [71], head and neck [67], esophageal and gastric cancer [65]. Negative *ANXA1* expression was also a poor prognostic indicator in pancreatic ductal adenocarcinoma, and showed a direct role in enhancing tumor aggressiveness in prostate cancer [72, 73]. From these evidences, *ANXA1* is suggested to act as a tumor suppressor gene. It has been implicated in the regulation of cell proliferation, differentiation, motility, trafficking, apoptosis, and tissue architecture [65]. In the presence of DNA damage, annexin A1 is translocated to the nucleus and functions as a stress protein or protective protein [74]. It also has been shown to be localized at the cell surface, playing an important role in myoblast and fibroblast migration and cytoskeleton reorganization, thus highlighting its role in cancer cell invasion and metastasis [75]. Additionally, gastric cell lines with low *ANXA1* expression showed higher potency for cell growth and invasion than that with high *ANXA1* expression via regulating *COX-2* (cyclooxygenase-2) expression [65]. Underlying molecular mechanism of reduced *ANXA1* expression in cancer has been yet undefined. Possible mechanisms include decreased transcription by genomic deletions, truncating mutations, or promotor hypermethylation [63], or altered posttranscriptional processing by miRNA targeting as we suggested in our results. This evidence is consistent with the study of Luthra and colleagues [28] which showed a significant inverse correlation of miR-196a2 with *ANXA1* mRNA levels in 12 cancer cell lines of esophageal, breast and endometrial origin, and that of Pin and his colleagues [14] that demonstrated miR-196a2 regulation of *ANXA1* expression in endothelial cells. Taken together, these findings along with ours suggest the potential role of *ANXA1* in cancer development and progression.

Another important finding in the present study was the *DFFA* and *PDCD4* decreased expression in all GI cancer patients. Several studies have demonstrated high and low expression of *DFFA* in a variety of human tumors [76]. *DFFA* expression was found to be down-regulated during the exponential phase of growth in several human colon cancer cell lines compared with human normal colon cells [23]. *DFFA* (-/-) mice were associated with tumor progression and severe genomic instability in colon epithelial cells [77]. Decreased expression was also correlated with poor prognosis in esophageal cancer patients [22]. However, it was frequently up-regulated in ovarian serous carcinoma and represented a marker of aggressive behavior and poor prognosis [78]. We presumed transcriptional deregulation or posttranscriptional silencing that caused this aberrant expression of *DFFA* in cancer samples. Zhang *et al*. [21] reported DFFA protein, but not mRNA, to be decreased by miR-145 in staurosporine-induced tumor cell apoptosis *in vitro*. Our data along with these studies highlight *DFFA* crucial role in cancer biology. The DNA fragmentation factor, DFFA forms a heterodimeric protein with DFFB. DFFA protein has dual functions within the DFF complex. It acts as “a specific molecular chaperone” that facilitates the proper DFFB folding, and on the other hand, it inhibits the growing cells DFFB nuclease activity [79]. Upon induction of apoptosis, the cytosolic factor DFFA is cleaved by caspase-3 or granzyme B, exposing the active sites on DFFB. Released DFFB is translocated to the outer surface of the nuclear envelope to trigger a downstream signal transduction pathway [80]. DFFB interacts directly with Histone H1, attains its DNA binding ability and promotes its nuclease activity, thus triggers nuclear DNA fragmentation and chromatin condensation during apoptosis [76].

The tumor suppressor *PDCD4* was down-regulated in all cancer patients enrolled in the study. Diminished *PDCD4* expression was previously reported in head and neck squamous cell carcinoma [80], lung cancer [81], bladder cancer [82], and colon adenocarcinoma [83] compared to normal tissues. PDCD4 is involved in cell apoptosis, neoplastic transformation, and tumor progression [84, 85]. PDCD4 exerts its function by binding to the eukaryotic translation initiation factor 4A1 and hindering mRNA translation initiation and capping [86]. PDCD4 could also influence different cellular transcriptional pathways in various tumors [85]. PDCD4 suppressed carbonic anhydrase II (CAII) in human embryonic kidney [87], delayed cell cycle transition from G1 to S phase in glioma cancer cell [88], inhibited colon cancer cell invasion through suppressing MAP4K1 [89], and phosphorylated the specificity protein transcription factors in colorectal cells [90], thus promoting essential events important in driving invasion and metastasis [82]. Depletion of *PDCD4* expression was shown to alter the normal DNA-damage response and to prevent damaged cells from undergoing apoptosis [91]. On the other hand, over-expression of *PDCD4* leads to decreased proliferation and increased apoptosis in glioblastoma-derived cell lines, while its down-regulation by miR-21 facilitates glioblastoma proliferation *in vivo* [92]. In transgenic mice, PDCD4 suppressed tumor promotion and progression to carcinoma [93]. Similar effects have also been reported in studies conducted in colon and HCC cells [89, 94, 95]. In addition, it was found to be preferentially expressed in tumor promotor-resistant cells and suppressed in promotion-sensitive cells undergoing neoplastic transformation [85].

Despite the present results revealed a significant upregulation of miR-196a2 and low expression levels of the speculated target genes (*ANXA1*, *DFFA*, *PDCD4*) in all groups of GI cancer patients, a significant negative correlation of miR-196a2 with its putative targets could not be drawn. This finding could be explained by (1) the current relative small sample size that warrants further larger cohort validation study, (2) due to other genetic and epigenetic factors that could modulate their association; like the role of RNA binding proteins that can affect the binding of miRNAs to their targets [96] or the involvement of other types of non-coding RNAs such as long non-coding RNAs, small interfering RNAs, small nucleolar RNAs, small nuclear RNAs, Piwi-interacting RNAs, and circular RNAs; all of which additionally could affect the binding of miRNAs to their targets and subsequently change the final correlation results between their expressions [97], (3) the fact that gene expression results from integrated cell responses and cross-talk between different signaling pathways [98] "a single gene could be targeted by multiple miRNAs and one miRNA may regulate transcription of many genes simultaneously, thus forming complex genetic circuits in human cancer" which we cannot infer direct relation from it, and (4) the interesting previous findings of Liu et al. that the putative targets of miRNA could be regulated by translation inhibition rather than degradation and subsequently this would leave mRNA levels unaffected, but reduce the gene of interest protein expression levels [99]. This later suggesion was in agreement with our findings that mir-196a over-expression levels were negatively correlated with annexin A1 protein expression (r = -0.738, *P*<0.001), but with no significant correlation with the *ANXA1* mRNA levels. This warrants the need of evaluation of the other genes protein levels in cancer and non- cancer tissues along with the mRNA levels for the gene of interest to identify such type of regulation.

In conclusion our preliminary study does confirm the association of miR-196a2 up-regulation with GI cancers in our population, suggesting its potential role as a prognostic biomarker in these types of cancer. We confirm that the current study is a preliminary one limited by (1) the relatively small sample size that warrant further larger scale and functional studied for results validation, (2) unavailability of patients' survival data that could be correlated with the miRNA or gene expressions to support their prognostic roles in the current GIT cancer cases, and (3) despite our findings suggested the putative role of miR-196a2 and the selected genes in GI cancers, uncovering more differentially expressed genes under the control of cancer-related miRNAs in an integrative model approach will further increase our detailed understanding of GI cancer biology. In this regard, we should mention the community resource project; The Cancer Genome Atlas (TCGA) which provides a new source of information describing tumor and matched normal tissues from cancer (33 types) with more than 11000 patients for novel biomarkers identification [100]. Using this emerging source for evaluation of the presence of differentially expressed miRNAs (DEMs) and genes (DEGs) in GIT tumors with survival analysis should be the logic next step to confirm our findings.

## Supporting information

S1 Table Association between the expression profile of microRNA and targets and the clinicopathological features in cancer patients. (PDF)

S2 Table The major biological process, molecular function, cellular components and KEGG pathways related to *ANXA1*, *DFFA* and *PDCD4*. (PDF)
