# Supplementary material for: Evaluation of miRNA-196a2 and apoptosis-related target genes: ANXA1, DFFA and PDCD4 expression in gastrointestinal cancer patients: A pilot study
Source: PLoS One. 2017 Nov 1;12(11):e0187310. doi: 10.1371/journal.pone.0187310 (PMC5665540; doi:10.1371/journal.pone.0187310)
Supplement: S1 Table — (PDF) [file pone.0187310.s001.pdf]

Supplementary Table 1 Association between expression profile of microRNA and targets and clinic-pathological features in cancer patients.

	**miR-196a2**	**Annexin A1**	**ANXA1**	**DFFA**	**PDCD4**
**Age**					
EC	0.762	1.00	0.914	0.352	0.914
GC	0.240	0.054	0.304	0.304	0.374
SIC	0.571	0.571	0.857	0.571	0.857
CRC	0537	0.223	0.570	0.639	0.264
**Gender**					
EC	0.517	0.833	0.517	0.067	0.117
GC	1.00	0.573	0.142	0.662	**0.029**
SIC	0.629	0.114	0.067	1.00	0.057
CRC	0.072	0.400	0766	0.533	0.533
**Tumor grade**					
EC	0.610	0.275	0.171	1.00	0.352
GC	0.536	0.492	0.126	0.290	0.596
SIC	0.145	0.237	0.398	0.627	0.440
CRC	**0.002**	**0.004**	0.300	0.914	0.806
**Tumor size**					
EC	1.00	0.610	0.762	0.171	0.352
GC	0.223	0.246	0.225	0.705	0.295
SIC	0.190	0.857	0.857	0.571	0.381
CRC	**0.037**	**0.051**	**0.020**	0.584	0.622
**LN infiltration**					
EC	0.400	1.00	0.200	0.600	0.800
GC	0.297	0.380	1.000	0.717	0.756
SIC	0.400	0.629	0.629	1.00	1.00
CRC	0.481	0.938	0.938	0.696	0.621
**TNM Stage**					
EC	0.548	0.690	0.222	0.421	0.548
GC	0186	0.939	0.353	0.738	0.644
SIC	0.292	0.481	0.475	0.211	0.309
CRC	**0.039**	**0.021**	0.075	0.521	0.732

P values are shown. Mann-Whitney and Kruskal Wallis tests were used. Bold data indicate $P < 0.05$. EC, esophageal cancer; GC, gastric carcinoma; SIC, small intestine cancer; CRC, colorectal; TNM, Tumor size, Lymph node, and Metastasis staging system.
