# Supplementary material for: Evaluation of miRNA-196a2 and apoptosis-related target genes: ANXA1, DFFA and PDCD4 expression in gastrointestinal cancer patients: A pilot study
Source: PLoS One. 2017 Nov 1;12(11):e0187310. doi: 10.1371/journal.pone.0187310 (PMC5665540; doi:10.1371/journal.pone.0187310)
Supplement: S2 Table — (PDF) [file pone.0187310.s002.pdf]

	A	B	C	D	E	F	G
1		**#pathway ID**	**pathway description**	**observed gene count**	**false discovery rate**	**matching proteins in your network (IDs)**	**matching proteins in your network (labels)**
2	1	GO.0016281	eukaryotic translation initiation factor 4F complex	4	4.79e-08	ENSP00000293831,ENSP00000326381,ENSP00000338020,ENSP00000364073	EIF4A1,EIF4A2,EIF4G1,EIF4G3
3	2	GO.0005829	cytosol	14	2.85e-06	ENSP00000225577,ENSP00000231487,ENSP00000280154,ENSP00000293831,ENSP00000307272,ENSP00000311032,ENSP00000326381,ENSP00000338020,ENSP00000344818,ENSP00000354558,ENSP00000359206,ENSP00000364073,ENSP00000366237,ENSP00000367454	BTRC,CASP3,DFFA,DFFB,EIF4A1,EIF4A2,EIF4G1,EIF4G3,MTOR,PDCD4,RPS6KB1,RPTOR,SKP1,UBC
4	3	GO.0005654	nucleoplasm	11	0.00134	ENSP00000225577,ENSP00000231487,ENSP00000271638,ENSP00000280154,ENSP00000307272,ENSP00000311032,ENSP00000344818,ENSP00000354558,ENSP00000359206,ENSP00000366237,ENSP000003674	BTRC,CASP3,DFFA,DFFB,MTOR,PDCD4,RPS6KB1,RPTOR,S100A11,SKP1,UBC
5	4	GO.0031931	TORC1 complex	2	0.00179	ENSP00000307272,ENSP00000354558	MTOR,RPTOR
6	5	GO.0031981	nuclear lumen	11	0.00421	ENSP00000225577,ENSP00000231487,ENSP00000271638,ENSP00000280154,ENSP00000307272,ENSP00000311032,ENSP00000344818,ENSP00000354558,ENSP00000359206,ENSP00000366237,ENSP00000367454	BTRC,CASP3,DFFA,DFFB,MTOR,PDCD4,RPS6KB1,RPTOR,S100A11,SKP1,UBC
7	6	GO.0032991	macromolecular complex	12	0.00608	ENSP00000231487,ENSP00000257497,ENSP00000293831,ENSP00000307272,ENSP00000311032,ENSP00000326381,ENSP00000338020,ENSP00000354558,ENSP00000359206,ENSP00000364073,ENSP00000366237,ENSP00000367454	ANXA1,BTRC,CASP3,DFFA,DFFB,EIF4A1,EIF4A2,EIF4G1,EIF4G3,MTOR,RPTOR,SKP1
8	7	GO.0044444	cytoplasmic part	14	0.0158	ENSP00000231487,ENSP00000280154,ENSP00000293831,ENSP00000302707,ENSP00000307272,ENSP00000311032,ENSP00000326381,ENSP00000338020,ENSP00000344818,ENSP00000354558,ENSP00000359206,ENSP00000364073,ENSP00000366237,ENSP00000367454	BTRC,CASP3,DFFA,DFFB,EIF4A1,EIF4A2,EIF4G1,EIF4G3,FPR1,MTOR,PDCD4,RPTOR,SKP1,UBC
9	8	GO.0019005	SCF ubiquitin ligase complex	2	0.0368	ENSP00000231487,ENSP00000359206	BTRC,SKP1
10	9	GO.0043234	protein complex	10	0.0385	ENSP00000231487,ENSP00000257497,ENSP00000293831,ENSP00000307272,ENSP00000311032,ENSP00000326381,ENSP00000338020,ENSP00000354558,ENSP00000359206,ENSP00000364073	ANXA1,BTRC,CASP3,EIF4A1,EIF4A2,EIF4G1,EIF4G3,MTOR,RPTOR,SKP1

	A	B	C	D	E	F	G
1		**pathway ID#**	**pathway description**	**observed gene count**	**false discovery rate**	**(IDs)matching proteins in your network**	**(labels)matching proteins in your network**
2	1	GO.0009057	macromolecule catabolic process	10	6.06e-06	ENSP00000231487,ENSP00000293831,ENSP00000311032,ENSP00000326381,ENSP00000338020,ENSP00000344818,ENSP00000354558,ENSP00000359206,ENSP00000366237,ENSP00000367454	BTRC,CASP3,DFFA,DFFB,EIF4A1,EIF4A2,EIF4G1,MTOR,SKP1,UBC
3	2	GO.0007166	cell surface receptor signaling pathway	12	1.66e-05	ENSP00000225577,ENSP00000231487,ENSP00000257497,ENSP00000293831,ENSP00000307272,ENSP00000311032,ENSP00000326381,ENSP00000338020,ENSP00000344818,ENSP00000354558,ENSP00000359206,ENSP00000364073	ANXA1,BTRC,CASP3,EIF4A1,EIF4A2,EIF4G1,EIF4G3,MTOR,RPS6KB1,RPTOR,SKP1,UBC
4	3	GO.0044265	cellular macromolecule catabolic process	9	1.66e-05	ENSP00000231487,ENSP00000293831,ENSP00000311032,ENSP00000326381,ENSP00000338020,ENSP00000344818,ENSP00000359206,ENSP00000366237,ENSP00000367454	BTRC,CASP3,DFFA,DFFB,EIF4A1,EIF4A2,EIF4G1,SKP1,UBC
5	4	GO.0051338	regulation of transferase activity	9	3.1e-05	ENSP00000231487,ENSP00000280154,ENSP00000302707,ENSP00000307272,ENSP00000311032,ENSP00000326381,ENSP00000344818,ENSP00000354558,ENSP00000359206	BTRC,CASP3,EIF4A2,FPR1,MTOR,PDCD4,RPTOR,SKP1,UBC
6	5	GO.0034097	response to cytokine	8	3.7e-05	ENSP00000225577,ENSP00000257497,ENSP00000293831,ENSP00000311032,ENSP00000326381,ENSP00000338020,ENSP00000344818,ENSP00000364073	ANXA1,CASP3,EIF4A1,EIF4A2,EIF4G1,EIF4G3,RPS6KB1,UBC
7	6	GO.0051726	regulation of cell cycle	9	3.7e-05	ENSP00000225577,ENSP00000231487,ENSP00000257497,ENSP00000280154,ENSP00000307272,ENSP00000311032,ENSP00000344818,ENSP00000354558,ENSP00000359206	ANXA1,BTRC,CASP3,MTOR,PDCD4,RPS6KB1,RPTOR,SKP1,UBC
8	7	GO.0007165	signal transduction	14	0.000351	ENSP00000231487,ENSP00000257497,ENSP00000271638,ENSP00000293831,ENSP00000302707,ENSP00000307272,ENSP00000311032,ENSP00000326381,ENSP00000338020,ENSP00000340191,ENSP00000344818,ENSP00000354558,ENSP00000359206,ENSP00000364073	ANXA1,BTRC,CASP3,EIF4A1,EIF4A2,EIF4G1,EIF4G3,FPR1,FPR2,MTOR,RPTOR,S100A11,SKP1,UBC
9	8	GO.0034655	nucleobase-containing compound catabolic process	6	0.000351	ENSP00000293831,ENSP00000311032,ENSP00000326381,ENSP00000338020,ENSP00000366237,ENSP00000367454	CASP3,DFFA,DFFB,EIF4A1,EIF4A2,EIF4G1
10	9	GO.0031929	TOR signaling	3	0.000399	ENSP00000225577,ENSP00000307272,ENSP00000354558	MTOR,RPS6KB1,RPTOR
11	10	GO.0006309	apoptotic DNA fragmentation	3	0.000426	ENSP00000311032,ENSP00000366237,ENSP00000367454	CASP3,DFFA,DFFB
12	11	GO.0006446	regulation of translational initiation	4	0.000426	ENSP00000225577,ENSP00000326381,ENSP00000338020,ENSP00000364073	EIF4A2,EIF4G1,EIF4G3,RPS6KB1
13	12	GO.0016032	viral process	7	0.000634	ENSP00000231487,ENSP00000293831,ENSP00000326381,ENSP00000338020,ENSP00000344818,ENSP00000359206,ENSP00000364073	BTRC,EIF4A1,EIF4A2,EIF4G1,EIF4G3,SKP1,UBC
14	13	GO.0000289	nuclear-transcribed mRNA poly(A) tail shortening	3	0.000752	ENSP00000293831,ENSP00000326381,ENSP00000338020	EIF4A1,EIF4A2,EIF4G1
15	14	GO.0030262	apoptotic nuclear changes	3	0.000844	ENSP00000311032,ENSP00000366237,ENSP00000367454	CASP3,DFFA,DFFB
16	15	GO.0043200	response to amino acid	4	0.000844	ENSP00000225577,ENSP00000307272,ENSP00000311032,ENSP00000354558	CASP3,MTOR,RPS6KB1,RPTOR
17	16	GO.0051704	multi-organism process	10	0.000887	ENSP00000225577,ENSP00000231487,ENSP00000293831,ENSP00000311032,ENSP00000326381,ENSP00000338020,ENSP00000344818,ENSP00000354558,ENSP00000359206,ENSP00000364073	BTRC,CASP3,EIF4A1,EIF4A2,EIF4G1,EIF4G3,MTOR,RPS6KB1,SKP1,UBC
18	17	GO.0042221	response to chemical	12	0.00128	ENSP00000257497,ENSP00000293831,ENSP00000302707,ENSP00000307272,ENSP00000311032,ENSP00000326381,ENSP00000338020,ENSP00000340191,ENSP00000344818,ENSP00000354558,ENSP00000359206,ENSP00000364073	ANXA1,BTRC,CASP3,EIF4A1,EIF4A2,EIF4G1,EIF4G3,FPR1,FPR2,MTOR,RPTOR,UBC
19	18	GO.0006417	regulation of translation	5	0.00134	ENSP00000225577,ENSP00000326381,ENSP00000338020,ENSP00000354558,ENSP00000364073	EIF4A2,EIF4G1,EIF4G3,MTOR,RPS6KB1
20	19	GO.0071310	cellular response to organic substance	9	0.0018	ENSP00000257497,ENSP00000293831,ENSP00000307272,ENSP00000326381,ENSP00000338020,ENSP00000344818,ENSP00000354558,ENSP00000359206,ENSP00000364073	ANXA1,BTRC,EIF4A1,EIF4A2,EIF4G1,EIF4G3,MTOR,RPTOR,UBC
21	20	GO.0010033	response to organic substance	10	0.00187	ENSP00000257497,ENSP00000293831,ENSP00000307272,ENSP00000311032,ENSP00000326381,ENSP00000338020,ENSP00000344818,ENSP00000354558,ENSP00000359206,ENSP00000364073	ANXA1,BTRC,CASP3,EIF4A1,EIF4A2,EIF4G1,EIF4G3,MTOR,RPTOR,UBC
22	21	GO.0006952	defense response	8	0.00235	ENSP00000225577,ENSP00000231487,ENSP00000257497,ENSP00000338020,ENSP00000340191,ENSP00000344818,ENSP00000354558,ENSP00000359206	ANXA1,BTRC,EIF4G1,FPR2,MTOR,RPS6KB1,SKP1,UBC
23	22	GO.0044700	single organism signaling	13	0.00235	ENSP00000231487,ENSP00000271638,ENSP00000293831,ENSP00000302707,ENSP00000307272,ENSP00000311032,ENSP00000326381,ENSP00000338020,ENSP00000340191,ENSP00000344818,ENSP00000354558,ENSP00000359206,ENSP00000364073	BTRC,CASP3,EIF4A1,EIF4A2,EIF4G1,EIF4G3,FPR1,FPR2,MTOR,RPTOR,S100A11,SKP1,UBC
24	23	GO.0008286	insulin receptor signaling pathway	4	0.00277	ENSP00000225577,ENSP00000307272,ENSP00000338020,ENSP00000354558	EIF4G1,MTOR,RPS6KB1,RPTOR

	A	B	C	D	E	F	G
1		pathway ID#	pathway description	observed gene count	false discovery rate	(IDs)matching proteins in your network	(labels)matching proteins in your network
25	24	GO.0007154	cell communication	13	0.0028	ENSP00000231487,ENSP00000271638,ENSP00000293831,ENSP00000302707,ENSP00000307272,ENSP00000311032,ENSP00000326381,ENSP00000338020,ENSP00000340191,ENSP00000344818,ENSP00000354558,ENSP00000359206,ENSP00000364073	BTRC,CASP3,EIF4A1,EIF4A2,EIF4G1,EIF4G3,FPR1,FPR2,MTOR,RPTOR,S100A11,SKP1,UBC
26	25	GO.0000288	nuclear-transcribed mRNA catabolic process, deadenylation-depe	3	0.00354	ENSP00000293831,ENSP00000326381,ENSP00000338020	EIF4A1,EIF4A2,EIF4G1
27	26	GO.0019221	cytokine-mediated signaling pathway	5	0.00394	ENSP00000293831,ENSP00000326381,ENSP00000338020,ENSP00000344818,ENSP00000364073	EIF4A1,EIF4A2,EIF4G1,EIF4G3,UBC
28	27	GO.0045859	regulation of protein kinase activity	6	0.00394	ENSP00000280154,ENSP00000302707,ENSP00000307272,ENSP00000311032,ENSP00000344818,ENSP00000354558	CASP3,FPR1,MTOR,PDCD4,RPTOR,UBC
29	28	GO.0006259	DNA metabolic process	6	0.00418	ENSP00000257497,ENSP00000311032,ENSP00000344818,ENSP00000354558,ENSP00000366237,ENSP00000367454	ANXA1,CASP3,DFFA,DFFB,MTOR,UBC
30	29	GO.0007169	transmembrane receptor protein tyrosine kinase signaling pathwa	6	0.00418	ENSP00000225577,ENSP00000307272,ENSP00000311032,ENSP00000338020,ENSP00000344818,ENSP00000354558	CASP3,EIF4G1,MTOR,RPS6KB1,RPTOR,UBC
31	30	GO.0060255	regulation of macromolecule metabolic process	13	0.00433	ENSP00000225577,ENSP00000231487,ENSP00000271638,ENSP00000280154,ENSP00000293831,ENSP00000302707,ENSP00000307272,ENSP00000326381,ENSP00000338020,ENSP00000344818,ENSP00000359206,ENSP00000364073,ENSP00000366237	BTRC,DFFA,EIF4A1,EIF4A2,EIF4G1,EIF4G3,FPR1,PDCD4,RPS6KB1,RPTOR,S100A11,SKP1,UBC
32	31	GO.0080090	regulation of primary metabolic process	13	0.0047	ENSP00000225577,ENSP00000231487,ENSP00000257497,ENSP00000271638,ENSP00000280154,ENSP00000302707,ENSP00000307272,ENSP00000326381,ENSP00000338020,ENSP00000344818,ENSP00000359206,ENSP00000364073,ENSP00000366237	ANXA1,BTRC,DFFA,EIF4A2,EIF4G1,EIF4G3,FPR1,PDCD4,RPS6KB1,RPTOR,S100A11,SKP1,UBC
33	32	GO.0032268	regulation of cellular protein metabolic process	9	0.00612	ENSP00000225577,ENSP00000231487,ENSP00000280154,ENSP00000302707,ENSP00000307272,ENSP00000326381,ENSP00000338020,ENSP00000344818,ENSP00000364073	EIF4A2,EIF4G1,EIF4G3,FPR1,PDCD4,RPS6KB1,RPTOR,SKP1,UBC
34	33	GO.0071900	regulation of protein serine/threonine kinase activity	5	0.00612	ENSP00000280154,ENSP00000302707,ENSP00000307272,ENSP00000311032,ENSP00000344818	CASP3,FPR1,PDCD4,RPTOR,UBC
35	34	GO.0031323	regulation of cellular metabolic process	13	0.0064	ENSP00000225577,ENSP00000231487,ENSP00000257497,ENSP00000271638,ENSP00000280154,ENSP00000302707,ENSP00000307272,ENSP00000326381,ENSP00000338020,ENSP00000344818,ENSP00000359206,ENSP00000364073,ENSP00000366237	ANXA1,BTRC,DFFA,EIF4A2,EIF4G1,EIF4G3,FPR1,PDCD4,RPS6KB1,RPTOR,S100A11,SKP1,UBC
36	35	GO.0045786	negative regulation of cell cycle	5	0.00674	ENSP00000280154,ENSP00000307272,ENSP00000311032,ENSP00000344818,ENSP00000354558	CASP3,MTOR,PDCD4,RPTOR,UBC
37	36	GO.0032869	cellular response to insulin stimulus	4	0.00686	ENSP00000225577,ENSP00000307272,ENSP00000338020,ENSP00000354558	EIF4G1,MTOR,RPS6KB1,RPTOR
38	37	GO.0007346	regulation of mitotic cell cycle	5	0.00726	ENSP00000225577,ENSP00000231487,ENSP00000257497,ENSP00000344818,ENSP00000359206	ANXA1,BTRC,RPS6KB1,SKP1,UBC
39	38	GO.0051437	positive regulation of ubiquitin-protein ligase activity involved in re	3	0.00776	ENSP00000231487,ENSP00000344818,ENSP00000359206	BTRC,SKP1,UBC
40	39	GO.0006413	translational initiation	4	0.00871	ENSP00000293831,ENSP00000326381,ENSP00000338020,ENSP00000364073	EIF4A1,EIF4A2,EIF4G1,EIF4G3
41	40	GO.0097194	execution phase of apoptosis	3	0.00903	ENSP00000311032,ENSP00000366237,ENSP00000367454	CASP3,DFFA,DFFB
42	41	GO.0032270	positive regulation of cellular protein metabolic process	7	0.00905	ENSP00000225577,ENSP00000231487,ENSP00000302707,ENSP00000307272,ENSP00000311032,ENSP00000344818,ENSP00000359206	BTRC,CASP3,FPR1,RPS6KB1,RPTOR,SKP1,UBC
43	42	GO.0045945	positive regulation of transcription from RNA polymerase III promoter	2	0.00905	ENSP00000307272,ENSP00000354558	MTOR,RPTOR
44	43	GO.0031145	anaphase-promoting complex-dependent proteasomal ubiquitin-d	3	0.0104	ENSP00000231487,ENSP00000344818,ENSP00000359206	BTRC,SKP1,UBC
45	44	GO.0051171	regulation of nitrogen compound metabolic process	11	0.0104	ENSP00000225577,ENSP00000271638,ENSP00000280154,ENSP00000307272,ENSP00000326381,ENSP00000338020,ENSP00000344818,ENSP00000354558,ENSP00000359206,ENSP00000364073,ENSP00000366237	BTRC,DFFA,EIF4A2,EIF4G1,EIF4G3,MTOR,PDCD4,RPS6KB1,RPTOR,S100A11,UBC
46	45	GO.0051716	cellular response to stimulus	13	0.0104	ENSP00000231487,ENSP00000257497,ENSP00000271638,ENSP00000293831,ENSP00000302707,ENSP00000307272,ENSP00000311032,ENSP00000326381,ENSP00000338020,ENSP00000340191,ENSP00000344818,ENSP00000354558,ENSP00000364073	ANXA1,CASP3,EIF4A1,EIF4A2,EIF4G1,EIF4G3,FPR1,FPR2,MTOR,RPTOR,S100A11,SKP1,UBC
47	46	GO.1901990	regulation of mitotic cell cycle phase transition	4	0.0107	ENSP00000231487,ENSP00000257497,ENSP00000344818,ENSP00000359206	ANXA1,BTRC,SKP1,UBC
48	47	GO.0071345	cellular response to cytokine stimulus	5	0.011	ENSP00000293831,ENSP00000326381,ENSP00000338020,ENSP00000344818,ENSP00000364073	EIF4A1,EIF4A2,EIF4G1,EIF4G3,UBC
49	48	GO.0032870	cellular response to hormone stimulus	5	0.0123	ENSP00000225577,ENSP00000257497,ENSP00000307272,ENSP00000338020,ENSP00000354558	ANXA1,EIF4G1,MTOR,RPS6KB1,RPTOR
50	49	GO.0051439	regulation of ubiquitin-protein ligase activity involved in mitotic cell	3	0.0123	ENSP00000231487,ENSP00000344818,ENSP00000359206	BTRC,SKP1,UBC

	A	B	C	D	E	F	G
1		pathway ID#	pathway description	observed gene count	false discovery rate	(IDs)matching proteins in your network	(labels)matching proteins in your network
51	50	GO.0051347	positive regulation of transferase activity	5	0.0135	ENSP00000231487,ENSP00000302707,ENSP00000307272,ENSP00000344818,ENSP00000359206	BTRC,FPR1,RPTOR,SKP1,UBC
52	51	GO.0048511	rhythmic process	4	0.016	ENSP00000231487,ENSP00000257497,ENSP00000344818,ENSP00000359206	ANXA1,BTRC,SKP1,UBC
53	52	GO.0071495	cellular response to endogenous stimulus	6	0.018	ENSP00000225577,ENSP00000257497,ENSP00000307272,ENSP00000338020,ENSP00000344818,ENSP00000354558	ANXA1,EIF4G1,MTOR,RPS6KB1,RPTOR,UBC
54	53	GO.0022402	cell cycle process	6	0.0198	ENSP00000225577,ENSP00000231487,ENSP00000307272,ENSP00000344818,ENSP00000354558,ENSP00000359206	BTRC,MTOR,RPS6KB1,RPTOR,SKP1,UBC
55	54	GO.0044772	mitotic cell cycle phase transition	4	0.0199	ENSP00000225577,ENSP00000231487,ENSP00000344818,ENSP00000359206	BTRC,RPS6KB1,SKP1,UBC
56	55	GO.0051348	negative regulation of transferase activity	4	0.0215	ENSP00000280154,ENSP00000311032,ENSP00000326381,ENSP00000344818	CASP3,EIF4A2,PDCD4,UBC
57	56	GO.0031399	regulation of protein modification process	7	0.0218	ENSP00000231487,ENSP00000280154,ENSP00000302707,ENSP00000307272,ENSP00000311032,ENSP00000344818,ENSP00000359206	BTRC,CASP3,FPR1,PDCD4,RPTOR,SKP1,UBC
58	57	GO.0002223	stimulatory C-type lectin receptor signaling pathway	3	0.0224	ENSP00000231487,ENSP00000344818,ENSP00000359206	BTRC,SKP1,UBC
59	58	GO.0050790	regulation of catalytic activity	8	0.0224	ENSP00000231487,ENSP00000280154,ENSP00000302707,ENSP00000307272,ENSP00000326381,ENSP00000344818,ENSP00000354558,ENSP00000359206	BTRC,EIF4A2,FPR1,MTOR,PDCD4,RPTOR,SKP1,UBC
60	59	GO.0006412	translation	4	0.0235	ENSP00000293831,ENSP00000326381,ENSP00000338020,ENSP00000364073	EIF4A1,EIF4A2,EIF4G1,EIF4G3
61	60	GO.0009628	response to abiotic stimulus	6	0.0251	ENSP00000225577,ENSP00000257497,ENSP00000307272,ENSP00000311032,ENSP00000344818,ENSP00000354558	ANXA1,CASP3,MTOR,RPS6KB1,RPTOR,UBC
62	61	GO.0031146	SCF-dependent proteasomal ubiquitin-dependent protein cataboli	2	0.026	ENSP00000231487,ENSP00000359206	BTRC,SKP1
63	62	GO.0045862	positive regulation of proteolysis	4	0.026	ENSP00000231487,ENSP00000311032,ENSP00000344818,ENSP00000359206	BTRC,CASP3,SKP1,UBC
64	63	GO.0071407	cellular response to organic cyclic compound	4	0.026	ENSP00000225577,ENSP00000257497,ENSP00000311032,ENSP00000359206	ANXA1,BTRC,CASP3,RPS6KB1
65	64	GO.0000086	G2/M transition of mitotic cell cycle	3	0.0315	ENSP00000231487,ENSP00000344818,ENSP00000359206	BTRC,SKP1,UBC
66	65	GO.0002768	immune response-regulating cell surface receptor signaling pathw	4	0.0315	ENSP00000231487,ENSP00000344818,ENSP00000354558,ENSP00000359206	BTRC,MTOR,SKP1,UBC
67	66	GO.0007623	circadian rhythm	3	0.0315	ENSP00000231487,ENSP00000344818,ENSP00000359206	BTRC,SKP1,UBC
68	67	GO.0051384	response to glucocorticoid	3	0.0318	ENSP00000225577,ENSP00000257497,ENSP00000311032	ANXA1,CASP3,RPS6KB1
69	68	GO.0010165	response to X-ray	2	0.033	ENSP00000257497,ENSP00000311032	ANXA1,CASP3
70	69	GO.0031326	regulation of cellular biosynthetic process	10	0.0333	ENSP00000225577,ENSP00000257497,ENSP00000271638,ENSP00000280154,ENSP00000307272,ENSP00000326381,ENSP00000338020,ENSP00000344818,ENSP00000359206,ENSP00000364073	ANXA1,BTRC,EIF4A2,EIF4G1,EIF4G3,PDCD4,RPS6KB1,RPTOR,S100A11,UBC
71	70	GO.0010468	regulation of gene expression	10	0.0346	ENSP00000225577,ENSP00000280154,ENSP00000293831,ENSP00000307272,ENSP00000326381,ENSP00000338020,ENSP00000344818,ENSP00000354558,ENSP00000359206,ENSP00000364073	BTRC,EIF4A1,EIF4A2,EIF4G1,EIF4G3,MTOR,PDCD4,RPS6KB1,RPTOR,UBC
72	71	GO.0009408	response to heat	3	0.0354	ENSP00000225577,ENSP00000307272,ENSP00000354558	MTOR,RPS6KB1,RPTOR
73	72	GO.0001662	behavioral fear response	2	0.0357	ENSP00000225577,ENSP00000338020	EIF4G1,RPS6KB1
74	73	GO.0010243	response to organonitrogen compound	5	0.039	ENSP00000257497,ENSP00000307272,ENSP00000311032,ENSP00000338020,ENSP00000354558	ANXA1,CASP3,EIF4G1,MTOR,RPTOR
75	74	GO.0090304	nucleic acid metabolic process	10	0.039	ENSP00000257497,ENSP00000293831,ENSP00000307272,ENSP00000311032,ENSP00000326381,ENSP00000338020,ENSP00000344818,ENSP00000354558,ENSP00000366237,ENSP00000367454	ANXA1,CASP3,DFFA,DFFB,EIF4A1,EIF4A2,EIF4G1,MTOR,RPTOR,UBC
76	75	GO.0043434	response to peptide hormone	4	0.0416	ENSP00000257497,ENSP00000307272,ENSP00000338020,ENSP00000354558	ANXA1,EIF4G1,MTOR,RPTOR
77	76	GO.1901701	cellular response to oxygen-containing compound	5	0.0416	ENSP00000225577,ENSP00000257497,ENSP00000307272,ENSP00000338020,ENSP00000354558	ANXA1,EIF4G1,MTOR,RPS6KB1,RPTOR
78	77	GO.0002684	positive regulation of immune system process	5	0.0445	ENSP00000231487,ENSP00000257497,ENSP00000344818,ENSP00000354558,ENSP00000359206	ANXA1,BTRC,MTOR,SKP1,UBC
79	78	GO.0000082	G1/S transition of mitotic cell cycle	3	0.0455	ENSP00000225577,ENSP00000231487,ENSP00000344818	RPS6KB1,SKP1,UBC
80	79	GO.0045934	negative regulation of nucleobase-containing compound metaboli	6	0.0458	ENSP00000271638,ENSP00000280154,ENSP00000326381,ENSP00000344818,ENSP00000359206,ENSP00000366237	BTRC,DFFA,EIF4A2,PDCD4,S100A11,UBC
81	80	GO.0007049	cell cycle	6	0.0476	ENSP00000225577,ENSP00000231487,ENSP00000307272,ENSP00000344818,ENSP00000354558,ENSP00000359206	BTRC,MTOR,RPS6KB1,RPTOR,SKP1,UBC
82	81	GO.0043066	negative regulation of apoptotic process	5	0.0478	ENSP00000257497,ENSP00000280154,ENSP00000311032,ENSP00000344818,ENSP00000366237	ANXA1,CASP3,DFFA,PDCD4,UBC
83	82	GO.0002682	regulation of immune system process	6	0.0495	ENSP00000231487,ENSP00000257497,ENSP00000311032,ENSP00000344818,ENSP00000354558,ENSP00000359206	ANXA1,BTRC,CASP3,MTOR,SKP1,UBC

	B	C	D	E	F	G
1	**#pathway ID**	**pathway description**	**observed gene count**	**false discovery rate**	**(IDs)matching proteins in your network**	**matching proteins in your network (labels)**
2	GO.0003743	translation initiation factor activity	4	0.000357	ENSP00000293831,ENSP00000326381,ENSP00000338020,ENSP00000364073	EIF4A1,EIF4A2,EIF4G1,EIF4G3
3	GO.0001156	TFIIIC-class transcription factor binding	2	0.001	ENSP00000307272,ENSP00000354558	MTOR,RPTOR
4	GO.0001030	RNA polymerase III type 1 promoter DNA binding	2	0.00113	ENSP00000307272,ENSP00000354558	MTOR,RPTOR
5	GO.0001031	RNA polymerase III type 2 promoter DNA binding	2	0.00113	ENSP00000307272,ENSP00000354558	MTOR,RPTOR
6	GO.0001032	RNA polymerase III type 3 promoter DNA binding	2	0.00113	ENSP00000307272,ENSP00000354558	MTOR,RPTOR
7	GO.0004982	N-formyl peptide receptor activity	2	0.00113	ENSP00000302707,ENSP00000340191	FPR1,FPR2
8	GO.0000339	RNA cap binding	2	0.0165	ENSP00000293831,ENSP00000364073	EIF4A1,EIF4G3
9	GO.0003723	RNA binding	7	0.0485	ENSP00000257497,ENSP00000280154,ENSP00000293831,ENSP00000326381,ENSP00000338020,ENSP00000344818,ENSP00000364073	ANXA1,EIF4A1,EIF4A2,EIF4G1,EIF4G3,PDCD4,UBC

	A	B	C	D	E	F	G
1		# pathway ID	pathway description	observed gene count	False discovery	matching proteins in your network (IDs)	matching proteins in your network (labels)
2	1	04150	mTOR signaling pathway	3	0.00172	ENSP00000225577,ENSP00000307272,ENSP00000354558	MTOR,RPS6KB1,RPTOR
3	2	05206	MicroRNAs in cancer	4	0.00172	ENSP00000280154,ENSP00000307272,ENSP00000311032,ENSP00000354558	CASP3,MTOR,PDCD4,RPTOR
4	3	05416	Viral myocarditis	3	0.00172	ENSP00000311032,ENSP00000338020,ENSP00000364073	CASP3,EIF4G1,EIF4G3
5	4	05205	Proteoglycans in cancer	4	0.00245	ENSP00000225577,ENSP00000280154,ENSP00000311032,ENSP00000354558	CASP3,MTOR,PDCD4,RPS6KB1
6	5	04210	Apoptosis	3	0.00299	ENSP00000311032,ENSP00000366237,ENSP00000367454	CASP3,DFFA,DFFB
7	6	04152	AMPK signaling pathway	3	0.00703	ENSP00000225577,ENSP00000307272,ENSP00000354558	MTOR,RPS6KB1,RPTOR
8	7	04910	Insulin signaling pathway	3	0.00818	ENSP00000225577,ENSP00000307272,ENSP00000354558	MTOR,RPS6KB1,RPTOR
9	8	03013	RNA transport	3	0.00946	ENSP00000326381,ENSP00000338020,ENSP00000364073	EIF4A2,EIF4G1,EIF4G3
10	9	04710	Circadian rhythm	2	0.00946	ENSP00000231487,ENSP00000359206	BTRC,SKP1
11	10	05150	Staphylococcus aureus infection	2	0.0261	ENSP00000302707,ENSP00000340191	FPR1,FPR2
12	11	05221	Acute myeloid leukemia	2	0.0276	ENSP00000225577,ENSP00000354558	MTOR,RPS6KB1
13	12	04350	TGF-beta signaling pathway	2	0.0492	ENSP00000225577,ENSP00000231487	RPS6KB1,SKP1
